# Impact of acne on quality of life in young Pakistani adults and its relationship with severity: A multicenter study

**DOI:** 10.12669/pjms.37.3.2819

**Published:** 2021

**Authors:** Shaheen Naveed, Sadia Masood, Atiya Rahman, Safia Awan, Saadia Tabassum

**Affiliations:** 1 Dr. Shaheen Naveed, MBBS, FCPS. Assistant Professor of Dermatology, Liquate National Hospital, Karachi, Pakistan; 2 Dr. Sadia Masood, MBBS, FCPS, MHPE. Assistant Professor of Dermatology, Department of Medicine, Aga Khan University Hospital, Karachi, Pakistan; 3 Dr. Atiya Rahman, MBBS, FCPS. Associate Professor of Dermatology, Combined Military Hospital Lahore, Pakistan; 4 Ms. Safia Awan, MSC. Senior Instructor, Research, Department of Medicine, Aga Khan University Hospital, Karachi, Pakistan; 5 Dr. Saadia Tabassum, MBBS, FCPS. Assistant Professor of Dermatology, Department of Medicine, Aga Khan University Hospital, Karachi, Pakistan

**Keywords:** Acne Vulgaris, Severity, Quality of life, Impairment

## Abstract

**Objectives:**

The current study aimed to determine the effect of acne vulgaris on quality of life of young adults and to assess the correlation between acne severity and impairment of quality of life (QOLI).

**Methods:**

In this multi-center cross-sectional study, 163 young adults with acne vulgaris were assessed for severity and quality of life impairment between December 2016 and May 2017 at three tertiary care hospitals. Cardiff Acne Disability Index (CADI) was used to assess the quality of life while the severity of acne measured by Global Acne Grading System (GAGS). Data were collected on standardized forms, with CADI, GAGS scores, and sociodemographic data and analyzed using SPSS version 9. The quantitative variables were presented as means, median and qualitative variables expressed as frequency and percentages. P-value ≤0.05 was considered as statistically significant.

**Results:**

Out of 163 patients enrolled in this study, the mean age was 21.6 ± 4.9 years and 124 were females and 39 males. In mild QOLI, 56 (87.5%) cases were affected with mild, 11 (13.4%) with moderate and 1 (1.5%) case had severe acne. While, in moderate QOLI, 8(12.5%) cases were of mild, 60 (73.2%) were moderate and 6 (35.3%) cases affected with severe acne. The severe impairment of QOL noted in 11 (13.4%) moderate and 10 (58.8%) severe acne cases. The relationship between sex was statistically significant, (P<0.001). The result showed significant correlation between severity of acne vulgaris and the quality of life impairment of these patients (P<0.001).

**Conclusion:**

This study showed significant correlation between acne vulgaris and quality of life impairment. Cardiff acne disability index has proven to be a reliable tool to assess the quality of life. It is recommended to be used routinely in dermatology clinics to provide tailored treatment to individuals because mild disease may be disproportionately distressing for patients.

## INTRODUCTION

Acne Vulgaris is a common skin problem, that involves more than 85% of adolescents and two-third of young adults.^1^ Acne exhibits global distribution and affects young people when they undergo maximum physical, psychological and social changes.^2^ The face is commonly involved, and the affected individuals may have interpersonal difficulties, decreased self-esteem, and increased prevalence of anxiety and depression.^3^,^4^ The repercussion of acne on quality of life (QOL) is an important element of the morbidity related to acne, and it is the prime consideration while deciding about the initiation of treatments. Literature reported that psychological, emotional and social problems in acne patients are as common as those described by patients with diabetes, epilepsy, asthma or arthritis.^5^,^6^

Currently, there are various acne-specific quality of life instruments exist, like, Acne Disability Index (ADI), the Dermatology-Specific Quality of Life (DSQL) questionnaire and the Cardiff Acne Disability Index (CADI).^4^,^7^ In the current study, we used the Cardiff Acne Disability Index (CADI), because of its simplicity, validity, and reliability.^8^ It is user-friendly five-item questionnaire devised in 1989.^4^,^9^ Few prior studies have studied the psychosocial impact and QOL of the acne vulgaris patients and addressed the relationship between acne severity and emotional distress.^1^,^2^,^10^ However, we need to gather further data in our context with specific cultural norms. We, therefore, identified that there is a need to study the correlation between psychosocial impact and the severity of acne vulgaris in patients, using a reliable and validated tool. It will provide each individual with proper treatment, since even mild diseases can be disproportionately distressing to someone’s life.

## METHODS

The study is a multi-center cross-sectional study conducted in three different hospitals, named, Liaquat National Hospital, Karachi, and Combined Military Hospital, Lahore, Aga Khan University Hospital, Karachi. This study was conducted after approval by all the three hospital’s ethical review committees (CMH, Lahore, LNH and AKUH, Karachi). The study objectives were to determine the effect of acne vulgaris on the QOL of young adults and whether a correlation exists between the impairment of quality of life (QOLI) and acne severity. In this study, young adults with acne vulgaris, reporting to skin clinics of the respective hospitals seen during the study period i.e., between December 2016 and May 2017 meeting inclusion criteria were invited to participate. All the patients with any other chronic skin condition, systemic illness, under any psychiatric treatment, on systemic steroids, or pregnant were excluded from the study. Written informed consent was taken from all the participants.

QOL was measured using the CADI, while acne severity was objectively assessed by Global Acne Grading Scoring (GAGS). CADI is a five-item questionnaire. Every question has four answers with a score of 0-3. The overall scores are determined by adding scores of all questions with a minimal score of 0 and a maximal score of 15. The 0 to Five score interprets the mild quality of life impairment, 6 to 10 translates moderate, and 11 to 15 indicates severe QOLI. GAGS is a measurable grading system, based on locations, and type of lesions. It divides the areas into 6 different regions. These regions are five on the face, while one on the upper back or chest. These regions give a factor to each region. The nose and chin are given a factor of one while each cheek and forehead are given a factor of two. In addition, a factor of three is for chest/upper back. Scores translated as, one for comedones, two for papules, three for pustules, and four for nodules. Every region score is addition of severity score multiplied by the area factor. The maximum score is 44 and the minimum is one. The one to18 score is reflected as mild, 19 to 30 moderate, and 31 to 38 as severe, while 39 and above as very severe. By taking a prevalence of 12% and precision of 5%, the calculated sample size is 163 patients with the help of WHO software for sample size calculation taking 95% confidence level.^11^ Non-probability convenience sampling was used to raise the sample.

Data were collected using specific standardized forms, with CADI, GAGS scores, and sociodemographic data. Data were analyzed using SPSS version 19. The quantitative variables presented as mean±SD and median while the qualitative variables expressed as frequency and percentages. T-test was used to compare the significance of results for quantitative variables. Chi-square test was used to assess the association between categorical variables. P-value ≤0.05 considered as statistically significant.

## RESULTS

The study population consisted of 163 patients. There were 124 (76.1%) females and 39 (23.9%) males. The mean age of the study population was 21.6 ± 4.9 years. The majority of the study participants were students (n=100), 34 were professionals and 29 were house makers. Out of 163 patients, 129 were single and 34 were married. 23 patients belonged to the upper class, 125 middle and 15 were from lower class. Regarding educational status 15 (9.02%) patients acquired education till primary level, 76 (46.62%) had secondary and 72 (44.17%) completed tertiary education. The overall mean GAGS score was 19.8 (±7.7, range 10–40); Mild, moderate, and severe acne presented in 64 (39.3%), 82 (50.3%), and 17 (10.4%) patients, respectively. Among the patients with mild acne, there were 24 males and 40 females. Among moderate group, there were 12 males (30.8%) and 70 females (56.5%). Only 3 males (7.7%) and 14 females (11.3%) presented with severe acne. The mean CADI score was 6.70 (± 3.4, range 0-15), (Table-I).

**Table-I:** Characteristics of study population (n=163).

Participant’s features	n	%
Age	21.6 ± 4.9	21[18-25]
***Gender:***		
Male	39	23.9
Female	124	76.1
***Marital status:***		
Single	129	79.1
Married	34	20.9
***Occupation:***		
House makers	29	17.8
Students	100	61.3
Professionals	34	20.9
***Socioeconomic status:***		
Upper class	23	14.1
Middle class	125	76.7
Lower class	15	9.2
***Education:***		
Primary	15	9.02
Secondary	76	46.62
Tertiary	72	44.17
***Severity; GAGS***		
Mild	64	39.3
Moderate	82	50.3
Severe	17	10.4
GAGS score	19.8 ± 7.7	21[13-25]
CADI* score	6.7 ± 3.4	6[4-10]

CADI*: Cardiff acne disability index

Among the study participants, 68 patients (41.7 %) had mild QOLI (42 Female: 61.8% and 26 Male: 38.2%). 74 patients (45.4%) had moderate QOLI (10 Male: 13.5%, 64 Female: 86.5%), while 21 patients (12.9%) presented with severe QOLI (3 Male: 14.3%), 18 Female: 86.7%). The females were found to be significantly affected and the relationship between sex was found to be statistically significant, (P<0.001). QOLI was not found to be statistically significant with the marital status, 59 (86.8%) single participants, and 9 (13.2%) married participants presented with mild impairment, while, 53 (71.6%) single and 21(28.4%) married were in moderate group. Severe QOLI was found in 4(19.0%) married and 17(81.0%) single patients. Regarding the association of QOLI with education, 5 (7.3%) primary, 30(44.1) secondary and 32(43.2) tertiary level educated participants possessed mild QOLI. Moderate impairment was noticed in5(6.7) primary, 32(43.2) secondary, and 37(50.0) tertiary level educators. The severe impairment was observed in 5(23.8) primary, 14(66.6) secondary, and 2(9.5) tertiary level participants. These values were statistically significant (P< 0.01).

The association of QOLI with occupation quite significant, 6 (8.8%) house makers, 43 (63.2%) students and 19 (27.9%) professionals experienced mild QOLI. On the other hand, moderate impairment was noticed in 18 (24.3%) house makers, 42 (56.8%) students, and 14 (18.9%) professionals. The severe impairment was observed in 5 (23.8%) house makers, 15 (71.4%) students, and 1 (4.8%) professional. These values were statistically significant (P< 0.05). Among the socioeconomic status, 7 (10.3%) upper class, 58 (85.3%) middle class, and 3(4.4%) lower-class patients presented with mild QOLI. While, 15 (20.3%) upper class, 51 (68.9%) middle class, and 8(10.8%) lower-class participants presented with moderate QOLI. The severe QOLI was found in 1 (4.8%) patient of the upper class, 16 (76.2%) middle class, and 4 (19.0%) of lower social class, Table-II.

**Table-II:** Quality of life impairment and its association with other covariates.

Study Variables	Quality of life impaired (CADI)			*p* value

	Mild; 0-5 n=68 (41.7%)	Moderate; 6-10 n=74(45.4%)	Severe; 11-15 n=21 (12.9%)	
Age, years	21.2 ± 4.7	22.4 ± 4.9	20.1 ± 4.9	0.09
***Gender***				
Male	26 (38.2)	10 (13.5)	3 (14.3)	0.001
Female	42 (61.8)	64 (86.5)	18 (85.7)	
***Marital status***				
Single	59 (86.8)	53 (71.6)	17 (81.0)	0.08
Married	9 (13.2)	21(28.4)	4 (19.0)	
***Education***				
Primary	5(7.3)	5(6.7)	5(23.8)	
Secondary	30(44.1)	32(43.2)	14(66.6)	0.01
Tertiary	33(48.5)	37(50.0)	2(9.5)	
***Occupation***				
Housewife	6 (8.8)	18 (24.3)	5 (23.8)	0.03
Student	43 (63.2)	42 (56.8)	15 (71.4)	
Professional	19 (27.9)	14 (18.9)	1 (4.8)	
***Socioeconomic status***				
Upper class	7 (10.3)	15 (20.3)	1 (4.8)	0.053
Middle class	58 (85.3)	51 (68.9)	16 (76.2)	
Lower Class	3(4.4)	8 (10.8)	4 (19.0)	

The study results showed a significant correlation between GAGS and QOLI (P<0.001). In mild impairment of QOLI, 56 (87.5%) cases were affected with mild acne, 11(13.4%) with moderate and 1(1.5%) case had severe acne. While, in moderate QOLI, 8(12.5%) cases were of mild acne, 60 (73.2%) were of moderate acne and 6 (35.3%) cases were affected with severe acne. The severe impairment of QOL was noted in 11(13.4%) cases of moderate and 10(58.8%) cases of severe acne, (Table-III).

**Table-III:** Association between CADI score and Severity of acne (GAGS Scores).

Association between CADI* and GAGS** scores				

Impairment of Quality of life CADI Score (6.7 ± 3.4)	Mild acne; (1-18) n=64	Moderate Acne; (18-30) n=82	Severe Acne; (31-38) n=17	*p* value
Mild (0-5)	56 (87.5%)	11 (13.4%)	1 (5.9%)	
Moderate (6-10)	8 (12.5%)	60 (73.2%)	6 (35.3%)	<0.001
Severe (11-15)	0	11 (13.4%)	10 (58.8%)	

CADI*: Cardiff acne disability index, GAGS**: Global Acne Grading Scoring

## DISCUSSION

Healthy skin has a vital role in socialization and enhancing self-confidence.^3^,^5^ There are instances where cosmetic disfigurements produce social disapproval and low self-esteem.^12^,^13^ The medical literature shows an important and complex correlation between skin disorders and psychosocial functioning states of these patients.^14^,^15^ Different studies from various countries assessed the impact of acne on quality of life.^1^,^8^,^13^ It is very difficult to compare these different studies because of the differences in settings, culture and characteristics of the study population. There are very few studies published to assess the effect of acne on quality of life in our own local context.^16^-^18^

The current study was conducted in tertiary care centers of two provinces of Pakistan to get a diverse sample. The study population consisted of young adults, Shams et al, also conducted the study on similar age group.^18^ The young adults are usually conscious about their appearances and youth of a country contributes a lot in the progress of the country. It is imperative to be cognizant of the potential detriments to their mental health and to address the issues proficiently.^19^ There is variation in mean age group of patients in different studies population and different factors like age, gender and culture of that specific area affects the QOL.^20^ A study conducted by Kokandi on adult female university students, with mean age of 24 years, did not find a correlation between acne severity and QOLI ; neither CADI score was associated with disease duration or age of patients.^21^

In the current study there were more females than males. It is because females are more conscious about their skin and seek dermatological consultation more frequently. It may also have attributed to more regular use of beauty creams for attaining a fair blemish free complexion, but ending up with increased chances of skin damage and acne. The sex distribution was similar to that seen by El-Hamd et al (Female 60% vs Male, 40%) and Shyam et al.^4^,^19^ On the other hand, Do JE et al found males predominance in their study.^22^ The current study demonstrated strong association between gender and QOLI. Females have severe acne than males. This finding is also similar to that seen by Ismail KH et al.^23^ These observations were not similar to study by El-Hamd et al who did not find any significant difference between sex and acne grades.^4^ The mean global acne grading system score in our study was higher than most of the previous studies.^23^ Shams et al had similar observations about acne grading.^18^ These results were not consistent with studies by El-Hamd et al, Shyam et al and Kokandi, where most of the patients presented with mild acne.^4^,^19^,^20^ The total mean CADI score in our study was 6.70, which highlights that the average study population experienced moderate QOLI.

In this study we found a strong association between acne severity and CADI scores. Our results were consistent with those seen by El-Hamd et al, who also found that QOLI is significantly related to severity of acne.^4^ Also Shyam et al and Ismail KH et al found similar correlation.^19^,^23^ Yap et al and another study from Hong Kong did not found such association between CADI and acne severity.^8^,^24^ These variations probably reflect the different racial and cultural contexts.^21^,^25^

The results of the current study can serve as an interface for improving the QOL evaluation criteria as well as the measures to be taken for acne patients to prevent them from stress and mental trauma. This study provides an insight about the importance of using an effective measurable validated tool to assess the quality of life impairment in acne patients. It contributes to the studies conducted earlier by addressing the effectiveness of using CADI in our own context and suggests that acne treatment has to be tailored not merely based on grading but also on QOLI. The quality of life issues needs to be sought and addressed in acne vulgaris patients through in-depth counseling and psychotherapy.

### Limitations of the study:

It was conducted only among adolescent acne patients and may not reflect the findings of whole population. There is a need for future studies comprising all age groups including all provinces with a relatively larger sample size to validate the results of current study.

## CONCLUSION

The possible impairment in quality of life in acne patients need to be vigilantly assessed and, should be appropriately addressed. CADI has proven to be a convenient, valid and reliable tool to assess the quality of life within minutes. We recommend it to be used routinely in dermatology outpatient clinics to provide tailored treatment to each individual, as even mild disease may be disproportionately distressing to someone’s life. The acne vulgaris management should be based on acne severity and degree of quality of life impairment and it would help to improve the confidence and self-esteem of these patients.

### Authors Contribution:

**SN:** First author contributed in the conception, design, and analysis of this article, revising it critically for important logical contents and will also be responsible for final approval of the version to be published

SM: Second author contributed significantly in concept of study, analysis of data, and he also revised it critically multiple times for any further change, she will also be responsible for final approval.

**AR:** Contributed in the concept, analysis and critical review of article

**SA:** Contributed in critical analysis of data and design of the article and also go through the manuscript to see any further change

**ST:** Contributed in concept, critical review & data analysis

All the authors critically revised the manuscript and contributed to the discussion. The final version of the paper was read and approved by all authors.
